# Supporting Care Transformation Through a Comprehensive Graduate Medical Education Curricular Program in a Department of Internal Medicine

**DOI:** 10.1007/s11606-024-08908-2

**Published:** 2024-07-09

**Authors:** Christopher Moriates, Gregory Wallingford, Emily Vinas, Holli Sadler, F. Hudson Parker, Robin Reister, Michael Pignone

**Affiliations:** 1 VA Greater Los Angeles Healthcare System, Los Angeles, CA USA; 2 Department of Medicine, UCLA, Los Angeles, CA USA; 3 Department of Internal Medicine, Dell Medical School at The University of Texas at Austin, Austin, TX USA; 4 Department of Medical Education, Dell Medical School at The University of Texas at Austin, Austin, TX USA; 5 Central Texas VA Clinic, Austin, TX USA; 6 Department of Medicine, Duke University, Durham, NC USA; 7 Margolis Institute for Health Policy, Duke University, Durham, NC USA

## Abstract

**Background:**

The imperative to train physicians in skills required to lead care transformation is increasingly recognized, yet few Graduate Medical Education (GME) programs exist to meet this need.

**Aim:**

Describe the development, outcomes, and lessons learned from a novel GME care transformation curricular program.

**Setting:**

Department of Internal Medicine (IM) at Dell Medical School at The University of Texas, Austin.

**Participants:**

Between 2020 and 2023, 33 IM residents and fellows completed training with participation in the Care Transformation program.

**Program Description:**

Department leadership developed a comprehensive educational and experiential program that included (1) Dell Medical School-wide Distinction in Care Transformation curriculum; (2) Primary Care Residency track with care transformation projects; (3) participation in the national Veterans Affairs Chief Resident in Quality and Safety program; and (4) Hospital Medicine Fellowship in Quality and Safety.

**Program Evaluation:**

Each trainee led a care transformation project spanning a variety of topics and settings. Graduates who responded to a follow-up survey (22 of 33 graduates) reported they used skills learned through the program in their current roles and these experiences better prepared them for fellowship and/or faculty positions.

**Discussion:**

The Care Transformation program provides real-world experiences and skillsets that are increasingly valuable in modern healthcare delivery.

## INTRODUCTION

There is a growing mandate to include health systems science (HSS) principles and skills within Graduate Medical Education (GME) to equip physicians to address the evolving needs of effective healthcare delivery.^1–3^ As health systems transform, physicians need to be engaged in the work of driving improvements that ensure better care outcomes and enhance health equity. These skills are particularly important in Departments of Internal Medicine (IM), which have large numbers of trainees and care for high proportions of the most vulnerable patients.

Although the American Medical Association’s Accelerating Change in Medical Education consortium has helped introduce HSS curricula into medical schools, few GME programs have been organized around the unifying framework of HSS and instead have focused more narrowly on quality improvement (QI) and patient safety.^4, 5^ One potential reason for this gap is that the Accreditation Council for GME core competency of systems-based practice does not include a comprehensive conceptual framework, leaving notable gaps in several HSS domains.^4, 6^

The Department of IM at Dell Medical School at The University of Texas at Austin was established in 2016 with a key goal of advancing care transformation and improving health equity in its community. As a component of that work, Department leadership sought to establish a comprehensive HSS program that included active participation in a Dell Med School-wide educational program for all GME trainees focused on care transformation. The Dell Med Department of IM program was unique because it incorporated multiple reinforcing efforts that collectively integrated training and experiential projects across all HSS domains. This report provides a description of the Dell Med Department of IM program along with outcomes and lessons learned over 7 years of implementation.

## SETTING AND PARTICIPANTS

In 2016, with the establishment of the new Dell Medical School, we began planning a care transformation program within the new Department of IM. The first resident physicians were accepted to the program in 2017. Primary training sites include a safety-net hospital, a Federally Qualified Health Center (FQHC) clinic and a Veterans Affairs (VA) clinic. Between 2020 and 2023, 33 residents and fellows from the Department of IM completed training with participation in the Care Transformation program.

## PROGRAM DESCRIPTION

Prior to 2016, Austin’s community-based IM residency program offered limited exposure to HSS. The Department of IM developed four major reinforcing components for its care transformation educational approach:

1. Participation in the Dell Med School-wide Distinction in Care Transformation Program for select residents and fellows;
2. A new VA-based Primary Care (PC) Residency track that participates in the Distinction in Care Transformation Program;
3. Participation in the national VA Chief Resident in Quality and Safety (CRQS) Program;
4. A new Hospital Medicine Fellowship in Quality and Safety.

### Dell Med School-Wide Distinction in Care Transformation Program

The Distinction in Care Transformation program at Dell Med was created in 2017 as a multidisciplinary, longitudinal GME program that includes foundational coursework and experiential learning through a mentored healthcare transformation project, designed to develop physician leaders who are prepared to lead change in an evolving healthcare system.

The IM track was introduced as a categorical program in the National Resident Matching Program, created by re-purposing existing categorical slots. The first cohort of IM residents participated alongside resident colleagues in pediatrics, women’s health, and general surgery. The program grew from three to five IM residents per year, and now also includes participation from the PC track residents (described below), fellows in hospital medicine and palliative medicine, and resident trainees from multiple other specialties across Dell Medical School.

#### Health Systems Science Curriculum

The didactic curriculum is overseen by a multidisciplinary team of medical educators, including Associate Chairs from several departments. Its design addresses each component from the framework of HSS,^7^ including education in high-value care, QI, interprofessional communication skills, healthcare policy and equity, leadership, implementation science, and systems thinking. In addition, the curriculum included an emphasis on design thinking taught by human-centered design experts. The formal components of the Distinction in Care Transformation program begin in the second year of residency with two week-long sessions of “Foundations Coursework” that includes the full cohort of distinction program residents and fellows across the medical school. Delivery formats include a blend of case-based learning, interactive workshops, lectures, and experiential learning delivered synchronously and asynchronously. Learning sessions are grouped by theme. For example, there were sessions on interprofessional communication skills, a workshop using the “Crucial Conversations” framework,^8^ as well as a TeamSTEPPS program.^9^ High-value care and QI are taught in interactive sessions by faculty experts, and were supplemented by a subset of online Dell Med “Discovering Value-Based Health Care Learning Modules,” which are freely available, interactive resources that teach principles and approaches to create value in healthcare.^10^ Health system leaders met with residents to discuss healthcare policy and financing. Participants are also assigned to shadow hospital and clinic leaders to gain a first-hand, one-on-one perspective of healthcare leadership, pitch project ideas, and generate high-level support for their work.

#### Care Transformation Projects and Mentorship

In addition to didactics, a key component of the program is mentored experience in applying care transformation. Participants in the Distinction Program in Care Transformation, in consultation with program leaders and coaches, may choose to either design a new mentored project or select to lead a component of an ongoing program. IM projects span the inpatient and outpatient settings, as well as the community. Trainees are matched with project mentors with diverse interests within the Department of IM. Due to institutional priorities, local faculty expertise, and relative ease of implementation, QI projects were emphasized. The program also provides data support, QI coaching, and some administrative support for preparing scholarly output. Residents may commit 1 month of dedicated time during each of the second and third years of residency to further advance their projects, as well as use time during other rotations, electives, and ambulatory blocks (for example, a dedicated QI half-day each ambulatory week).

While each resident has a primary faculty mentor with specific content expertise, the resident cohorts are also coached by two Distinction Program leaders from within the Department, which ensure continued engagement. Each quarter, the Distinction Program leadership hosts a check-in dinner to build community among IM participants, discuss their projects, provide coaching, and review HSS-related literature. Participants are also required to submit progress reports twice at a regular cadence, which promotes accountability and triggers mentorship engagement and support in moving projects forward and ensuring that key program goals are met. The Distinction in Care Transformation Program concludes in May of the final year with presentations at Dell Med’s GME Research Day.

### A New VA-Based Primary Care Residency Track

In 2018, the Department of IM, in partnership with Central Texas VA leadership, created a separate PC residency track based at the Austin VA with four residents per class. Participation in the Distinction in Care Transformation program, including both the coursework and a care transformation project, is built into this residency track. PC track residents are encouraged to develop projects based in the outpatient setting, after identifying quality gaps or areas in need of improvement in their VA clinic practice. VA clinical faculty and the CRQS provide hands-on mentorship of these projects. Department of IM leadership instituted monthly Ambulatory QI Faculty Mentor meetings, which introduced a mechanism for ensuring continued progress and faculty development to drive projects.

### Participation in the National VA Chief Resident in Quality and Safety Program

In 2017, the Department of IM and the Central Texas VA, which is one of two ambulatory teaching sites for the Dell Med IM residency, applied for and received an ambulatory-focused CRQS position.^11^ The CRQS participates in the national VA curriculum for QI training, as well as in monthly Ambulatory QI Leadership meetings where Dell Med faculty mentors and coaches provide feedback and instruction about ongoing resident-led QI projects. The CRQS also helps lead the outpatient-based QI curricula for all residents. The CRQS serves as the primary lead for a QI project and provides direct guidance for resident-led ambulatory QI projects at the Austin VA IM Clinic. The CRQS works closely with the CRQS faculty mentor and the PC Track Program Director to assimilate into their role as a departmental leader for quality and safety.

### A Hospital Medicine Fellowship in Quality and Safety

In 2019, we introduced a Hospital Medicine Fellowship in Quality and Safety as an integrated 1-year program designed to provide post-graduates with clinical, academic, and administrative training in hospital medicine, with an emphasis on QI, patient safety, and health equity in our safety-net teaching hospital. Fellows dedicate approximately 50% of their time to inpatient clinical work as an attending physician and 50% to experiential work leading care transformation and patient safety projects, participating in relevant hospital committees and programs, and attending faculty development sessions. As part of the fellowship, trainees also participate in the Distinction in Care Transformation Program. Palliative Care fellows were also integrated into the Distinction in Care Transformation Program in 2020.

## PROGRAM EVALUATION

To assess program impact, we surveyed all IM graduates of the Distinction in Care Transformation Program, the CRQS program, the PC Residency Track, and the Hospital Medicine Fellowship in Quality and Safety in September 2023.

Between 2020 and 2023, 14 IM resident physicians graduated from the Distinction in Care Transformation Program; eight residents graduated from the PC Track; three Hospital Medicine Fellows in Quality and Safety and five Palliative Care Fellows graduated; and three CRQS’s graduated. Each of these participants led or co-led a care transformation project, across a variety of topic areas and settings (Table 1).

**Table 1:** Graduate Medical Education Trainee-Led Care Transformation Projects

Year of graduation	Training position	Project area
2020	Internal Medicine Distinction Track in Care Transformation Resident	Initiation of buprenorphine treatment and bridging to outpatient therapy for hospitalized patients with opioid use disorder^16–18^
2020	Internal Medicine Distinction Track in Care Transformation Resident	Women in IM Peer Mentorship Program
2020	Internal Medicine Distinction Track in Care Transformation Resident	Interprofessional education and improving discharge workflow
2020	VA Chief Resident of Quality and Safety	Multiple projects, including improving lung cancer screening at the VA
2020	Hospital Medicine Fellow in Quality and Safety	QI/safety handbook; patient safety case reviews; high-value care education^19^
2021	Internal Medicine Distinction Track in Care Transformation Resident	1. A systematic effort to screen for and address health-related social needs for patients admitted with COVID-19^20^ 2. Increasing rates of daily weight measurements for CHF patients on the 6th floor at DSMC
2021	Internal Medicine Distinction Track in Care Transformation Resident	Addressing kidney disease educational gaps for Hispanic immigrants receiving emergency-only dialysis^21^
2021	Internal Medicine Distinction Track in Care Transformation Resident	Creation of a Lung Cancer Screening Program at the VA Resident Clinic
2021	Internal Medicine Primary Care Track Resident	Evaluating changes in influenza vaccine hesitancy in an FQHC population during the COVID-19 pandemic
2021	Internal Medicine Primary Care Track Resident	Evaluating changes in influenza vaccine hesitancy in an FQHC population during the COVID-19 pandemic
2021	VA Chief Resident of Quality and Safety	Transition to telehealth at the VA during the pandemic
2021	Hospital Medicine Fellow in Quality and Safety	Alcohol use disorder program for hospitalized patients
2021	Palliative Care Fellow	Structured debriefs to address clinician emotions after cardiopulmonary arrest
2022	Internal Medicine Distinction Track in Care Transformation Resident	Patient health identity management utilizing a blockchain solution^22^
2022	Internal Medicine Distinction Track in Care Transformation Resident	Implementing a PENICILLIN allergy delabelling program
2022	Internal Medicine Distinction Track in Care Transformation Resident	Transitions of Care initiative working with the Design Institute for Health
2022	Internal Medicine Distinction Track in Care Transformation Resident	Improving nursing communication with IM residents
2022	Internal Medicine Primary Care Track Resident	Increasing utilization of SGLT-2 inhibitors in veterans with diabetes and chronic kidney disease
2022	Internal Medicine Primary Care Track Resident	Increasing safe utilization of SGLT-2 inhibitors in patients with type II diabetes and CKD 3A/3B in the outpatient setting
2022	VA Chief Resident of Quality and Safety	Screening for hyperaldosteronism in patients with secondary hypertension
2022	Palliative Care Fellow	Opportunities for improving outpatient palliative medicine curriculum
2022	Palliative Care Fellow	PPE Portraits During COVID-19
2023	Internal Medicine Distinction Track in Care Transformation Resident	Kidney screening and community health workers^23^
2023	Internal Medicine Distinction Track in Care Transformation Resident	Alcohol use disorder screening and treatment in inpatient management
2023	Internal Medicine Distinction Track in Care Transformation Resident	Utilization of pharmacist review of discharge medication reconciliation to improve patient outcomes
2023	Internal Medicine Distinction Track in Care Transformation Resident	Increasing completion of medical power of attorney documentation in the cardiology wards at a large tertiary referral hospital
2023	Internal Medicine Primary Care Track Resident	Improving nursing confidence and partnership with LGBTQ + Veterans through inclusivity and education
2023	Internal Medicine Primary Care Track Resident	Improving end-of-life planning in primary care
2023	Internal Medicine Primary Care Track Resident	Can we do sleep studies for cardiac patients admitted to the silver team?
2023	Internal Medicine Primary Care Track Resident	Equipping residents for end-of-life conversations in primary care
2023	VA Chief Resident of Quality and Safety	Multiple projects including improving mammogram screening, SGTL2i use for CHF, FIB-4 score for fatty liver disease screening, end-of-life planning in primary care
2023	Palliative Care Fellow	Implementing music therapy strategies in a setting of limited resources
2023	Palliative Care Fellow	Commit to Sit: a low-cost intervention to decrease barriers to sitting during inpatient patient interactions

In addition to leading projects as outlined in Table 1, the second cohort (2018–2021) of residents also stepped up in 2020 to lead and meaningfully contribute to efforts to screen for and address health-related social needs for patients with COVID-19,^12^ improve language-concordant communication for patients admitted with COVID-19,^13^ and provide additional direct care for patients in the ICU. These efforts were contributors to our high-level performance in limiting mortality from COVID-19 in 2020–2021.^14^

Thus far, all participants presented their projects locally at Dell Med GME Research Day, at least 16 have presented at national meetings, and six have published peer-reviewed articles related to their project. Sixteen participants have been selected as Chief Residents, and 18 were accepted to fellowships. Many graduates commented that they felt the skills and experiences they gained in the Distinction Program in Care Transformation impacted their career paths and increased their competitiveness for fellowship and/or faculty positions (Table 2).

**Table 2:** Feedback from Graduates About Impact of the Care Transformation Program

**Themes from responses / Example quotes**	
**How graduates are applying skills from the program in their current roles**	
Leading and teaching quality improvement initiatives	“I have applied the skills I learned regarding QI in designing multiple of my own QI projects in fellowship. I have also applied my knowledge of how to apply to and work with the IRB…” “The skills learned during my CRQS year allowed me to lead QI initiatives, mentor residents and developed my own skills in QI work…These skills have been an asset as an academic hospitalist and will serve me throughout my career.” “I am a faculty member for one of the residency QI cohorts and we just started a project! I hope to also participate in a [root-cause analysis] and other QI projects at the VA.” “Discussing my residents' QI projects and resources they could use to make their projects and ideas a reality.” “Run QI projects with residents, teach QI, use skills for QI and patient safety.”
Understanding health systems and change management	“Understanding healthcare system processes and navigating them to implement changes.” “Understanding health systems, payor structures, change management has allowed me to make more informed decisions on improvement and changes in care quality.” “…I also feel more confident in designing a project that will be effective through understanding change management better (appropriate stakeholders, establishing buy in at leadership level etc.).”
Applying specific skills and insights from project experience	“Importance of qualitative interviews. Learning how systems work when establishing a pilot program.” “Value-based care is a topic that comes up frequently in the private medicine world. Avoiding unnecessary testing, discouraging treatments such as unnecessary antibiotics, and avoiding unnecessary consults that might delay discharge is something you need to keep in mind every day.” “I’ve applied human centered design principles alongside QI projects.” “Presentation skills, leading meetings, planning a project with a PDSA cycle, building a team…all very applicable as I’ve transitioned to academic faculty.”
Collaborating with diverse interprofessional teams	“Through my interprofessional education elective and subsequent project in discharge workflow, I feel I am an effective hospitalist and was more than prepared with skills to work with non-physician staff at the end of residency.” “…Working on my distinctions project with a large, multifaceted team (clinicians, designers, admin, etc.) has also translated well for my current role/work with PIs, project managers, data analysts, and health systems leaders at [large academic program].” “Interdisciplinary collaboration across the healthcare spectrum.”
Advancing their projects and related scholarship after graduation	“Built important relationships with key mentors, worked with multidisciplinary teams to change overall culture and behavior, able to continue working on the same project and receive a grant, presented in numerous national venues and have had invited talks related to this project.” “I continue to present work of the B-Team and apply QI research skills and health systems science knowledge to navigate my current research in addiction.”
**Open-ended feedback from graduates about the care transformation program**	
Empowering graduates to lead health systems improvement	“It was an integral part of residency training and I became invested as a teacher and systems-improver…” **“**I think it overall was a very positive influence on my medical training and I will use these skills throughout my entire career. I feel more confident in approaching higher level positions and feel that I understand the world of QI and why it’s important so much better than before this program. It also really stimulated my interest in research, as I had very little research in medical school.” **“**My experience and learning about health care value, health system structures, and healthcare design completely changed my view of healthcare and how we change it for the future. I use those principles to help patients and projects to improve health care quality.” **“**The Distinction Program was an invaluable learning experience that taught me how to translate passion into action, and beyond that into sustainable development of programs that help our patients and our community. Faculty mentors were available at every step of the way to offer guidance and feedback. I would not have otherwise learned these skills and been able to put them into practice in real time as a fellow… “
Increased competitiveness for subsequent academic positions	“Experience in quality improvement and designing projects was something that many institutions were interested in while I was interviewing for academic positions.”
Attracted them to the residency program	“I chose to complete residency training at Dell Medical School at the University of Texas specifically for the additional project-based support offered by the Distinction Program in Care Transformation.” “… many say it was/is a big draw of the program.”

Twenty-two of 33 graduates (67%) completed the survey. Nearly all graduates (21 of 22 respondents, 95%) reported they used skills they learned through the Distinction in Care Transformation program and/or CRQS experience. Descriptions of how they have applied these skills are provided in Table 2.

## DISCUSSION

The Care Transformation program provides real-world experience, a focus on health equity, and a HSS skillset that is increasingly valuable in modern healthcare delivery. In addition, residents produce scholarly output with national presentations and publications related to their work. They apply their new skills in future positions as chief residents, clinical fellows, faculty, and practicing physicians.

While the program represents significant investment in faculty time, support, and infrastructure, components of this approach have already been adopted elsewhere, such as a similar “Health Systems Science Distinction Track” that has subsequently been introduced at UT Health San Antonio/Long School of Medicine.^15^ One key for success of the program was leveraging existing, under-deployed resources, such as using national VA programs to support the CRQS and primary care residency, as well as financing the hospital medicine fellow through clinical funds. For GME programs that cannot make as big of an investment, components can be integrated into already existing infrastructure for QI projects, resident research, and education. For example, GME-wide Grand Rounds or similar venues can be used to deliver foundational HSS content to trainees.

Lessons learned from our experience underline the importance of creating multiple reinforcing components (e.g., the school-wide educational program, a CRQS, and a hospital medicine fellowship program) and setting care transformation as an organizational priority for the Department, with active involvement from the Chair and Associate Chair. Another key learning was the importance of a dedicated program leader who served as a coach through regular informal and formal check-in meetings, ensuring continued support and professional development in addition to a traditional project mentor.

The Care Transformation programs across the Department of IM at Dell Medical School have set a foundation for creating real-world application of HSS within GME education and provide lessons for other programs facing the imperative to include this emerging competency within clinical training.
